# Astrocytes exhibit diverse Ca^2+^ changes at subcellular domains during brain aging

**DOI:** 10.3389/fnagi.2022.1029533

**Published:** 2022-10-28

**Authors:** Fusheng Ding, Shanshan Liang, Ruijie Li, Zhiqi Yang, Yong He, Shaofan Yang, Qingtian Duan, Jianxiong Zhang, Jing Lyu, Zhenqiao Zhou, Mingzhu Huang, Haoyu Wang, Jin Li, Chuanyan Yang, Yuxia Wang, Mingyue Gong, Shangbin Chen, Hongbo Jia, Xiaowei Chen, Xiang Liao, Ling Fu, Kuan Zhang

**Affiliations:** ^1^Britton Chance Center for Biomedical Photonics, Wuhan National Laboratory for Optoelectronics, Huazhong University of Science and Technology, Wuhan, China; ^2^MoE Key Laboratory for Biomedical Photonics, School of Engineering Sciences, Huazhong University of Science and Technology, Wuhan, China; ^3^Brain Research Center and State Key Laboratory of Trauma, Burns, and Combined Injury, Third Military Medical University, Chongqing, China; ^4^Advanced Institute for Brain and Intelligence and School of Physical Science and Technology, Guangxi University, Nanning, China; ^5^Brain Research Instrument Innovation Center, Suzhou Institute of Biomedical Engineering and Technology, Chinese Academy of Sciences, Suzhou, China; ^6^Combinatorial NeuroImaging Core Facility, Leibniz Institute for Neurobiology, Magdeburg, Germany; ^7^Guangyang Bay Laboratory, Chongqing Institute for Brain and Intelligence, Chongqing, China; ^8^Center for Neurointelligence, School of Medicine, Chongqing University, Chongqing, China

**Keywords:** astrocyte, Ca^2+^ transients, branches, branchlets and leaflets, endfeet, aging

## Abstract

Astrocytic Ca^2+^ transients are essential for astrocyte integration into neural circuits. These Ca^2+^ transients are primarily sequestered in subcellular domains, including primary branches, branchlets and leaflets, and endfeet. In previous studies, it suggests that aging causes functional defects in astrocytes. Until now, it was unclear whether and how aging affects astrocytic Ca^2+^ transients at subcellular domains. In this study, we combined a genetically encoded Ca^2+^ sensor (GCaMP6f) and *in vivo* two-photon Ca^2+^ imaging to determine changes in Ca^2+^ transients within astrocytic subcellular domains during brain aging. We showed that aging increased Ca^2+^ transients in astrocytic primary branches, higher-order branchlets, and terminal leaflets. However, Ca^2+^ transients decreased within astrocytic endfeet during brain aging, which could be caused by the decreased expressions of Aquaporin-4 (AQP4). In addition, aging-induced changes of Ca^2+^ transient types were heterogeneous within astrocytic subcellular domains. These results demonstrate that the astrocytic Ca^2+^ transients within subcellular domains are affected by aging differently. This finding contributes to a better understanding of the physiological role of astrocytes in aging-induced neural circuit degeneration.

## Introduction

Astrocytes are distributed throughout the brain in a grid-like manner and form a dense network of interactions with neurons, other glial cells, and blood vessels (Kosaka and Hama, 1986; Khakh and Sofroniew, 2015; Liu et al., 2020; Descalzi, 2021). These electrically non-excitable astrocytes use Ca^2+^ signals as a substrate for their excitability to communicate with the surrounding milieu (Jianxiong Zhang and Chen, 2017; Semyanov, 2019; Semyanov and Verkhratsky, 2021). Astrocytic Ca^2+^ elevations (transients) can influence the activities of surrounding neurons by releasing gliotransmitters (Araque et al., 2014), regulating K^+^ uptake (Wang et al., 2012), and controlling local blood flow (Otsu et al., 2015). Therefore, spatially and temporally controlled Ca^2+^ signals represent a major component of ‘astrocytic languages’ which mediate learning and memory (Zhang et al., 2021; Wu and Gao, 2022). More importantly, astrocytic Ca^2+^ transients occur more frequently following CNS injuries and are abnormally increased in regions of amyloid deposition in murine models of Alzheimer’s disease (Shigetomi et al., 2019; Verkhratsky, 2019), indicating that astrocytic Ca^2+^ signaling is highly adaptable and changeable. Additional studies have demonstrated that multiple patterns of astrocytic Ca^2+^ transients determine multiple states of neuronal networks (Semyanov, 2019). As such, the pathological changes in astrocytic Ca^2+^ transients contribute to astrocytic pathology associated with deficient neuroprotection or failure in glial homeostatic support (Nanclares et al., 2021).

**FIGURE 1:**
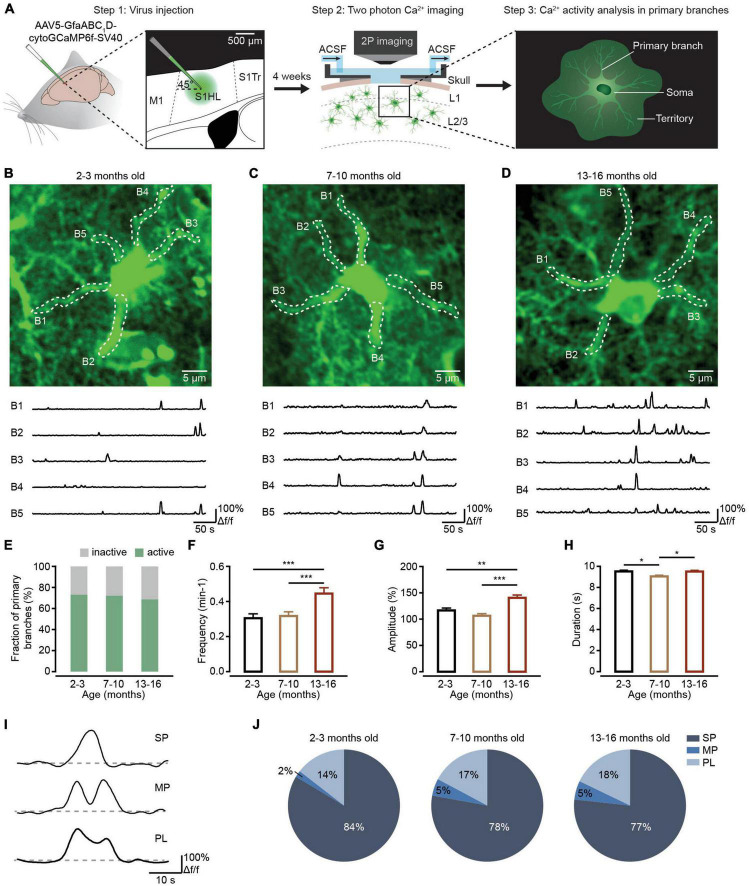
Ca^2+^ transient changes within primary branches of cortical astrocytes during brain aging. **(A)** Experimental flow diagram. Left: AAV5-GfaABC_1_D-cytoGCaMP6f-SV40 was injected into the S1 area. Middle: *in vivo* two-photon Ca^2+^ imaging was performed on the cortical astrocytes 4 weeks after viral injection. Right: *in vivo* Ca^2+^ transients were analyzed in primary astrocyte branches. **(B–D)** Top, representative two-photon images of GCaMP6f-labeled astrocytes in the 2–3-month-old (**B**; imaging depth, 110 μm), 7–10-month-old (**C**; imaging depth, 107 μm), and 13–16-month-old groups (**D**; imaging depth, 80 μm). Primary branch ROIs are outlined using white dashed lines. Below are example traces of spontaneous Ca^2+^ transients from each ROI. **(E)** The fraction of active primary branches within the 2–3-month-old (*n* = 183 ROIs from six mice), 7–10-month-old (*n* = 197 ROIs from nine mice), and 13–16-month-old groups (*n* = 169 ROIs from six mice); all groups: χ^2^ = 0.9739, *P* = 0.6145, χ^2^-test. **(F)** Bar graphs summarizing the frequencies of Ca^2+^ transients within all active branches of the 2–3 month-old (*n* = 129 ROIs from six mice), 7–10-month-old (*n* = 141 ROIs from nine mice), and 13–16-month-old (*n* = 110 ROIs from six mice) group. The 2–3-month-old group versus the 7–10 month-old group: *P* = 1.000; the 2–3-month-old group versus the 13–16-month-old group: *P* = 0.0003; the 7–10 month-old group versus the 13–16-month-old group: *P* = 0.0005; ***P < 0.001, Kruskal–Wallis test followed by Dunn’s multiple comparisons test. **(G)** Bar graphs summarizing the amplitudes of Ca^2+^ transients within active primary branches of the 2–3-month-old (*n* = 444 Ca^2+^ events from six mice), 7–10-month-old (*n* = 432 Ca^2+^ events from nine mice), and 13–16-month-old groups (*n* = 548 Ca^2+^ events from six mice). The 2–3-month-old group versus the 7–10-month-old group: *P* = 0.6555; the 2–3-month-old group versus the 13–16-month-old group: *P* = 0.0026; 7–10 months old group versus 13–16 months old group: *P* = 1.30 × 10^– 5^; ***P < 0.001,**P < 0.01, Kruskal–Wallis test followed by Dunn’s multiple comparisons test. **(H)** Bar graphs summarizing the durations of Ca^2+^ transients within active primary branches of the 2–3-month-old (*n* = 453 Ca^2+^ events from six mice), 7–10-month-old (*n* = 444 Ca^2+^ events from nine mice), and 13–16-month-old groups (*n* = 562 Ca^2+^ events from six mice). The 2–3 month-old group versus the 7–10-month-old group: *P* = 0.0173; the 2–3-month-old group versus 13–16-month-old group: *P* = 1.0000; the 7–10-month-old group versus the 13–16-month-old group: *P* = 0.0361; *P < 0.05, Kruskal–Wallis test followed by Dunn’s multiple comparisons test. **(I)** Example Ca^2+^ traces of the 3 peak types: singlepeaks, multipeaks, and plateaus. **(J)** Proportions of different peak types of Ca^2+^ transients within astrocytic branches of the 2–3-month-old (*n* = 469 Ca^2+^ events from six mice), 7–10-month-old (*n* = 465 Ca^2+^ events from nine mice) and 13–16-month-old groups (*n* = 588 Ca^2+^ events from six mice). All groups: χ^2^ = 12.1912, *P* = 0.0160; the 2–3-month-old group versus the 7–10-month-old group: SP: *P* = 0.0791, MP: *P* = 0.0330, PL: *P* = 0.7728; the 2–3 month-old group versus the 13–16-month-old group: SP: *P* = 0.0170, MP: *P* = 0.0096, PL: *P* = 0. 4278; the 7–10-month-old group versus the 13–16-month-old group: SP: *P* = 1.0000, MP: *P* = 1.0000, PL: *P* = 1.0000. χ^2^-test followed by Bonferroni correction. All data are shown as the mean ± SEM. SP, singlepeaks; MP, multipeaks; PL, plateaus; B, branch.

**FIGURE 2:**
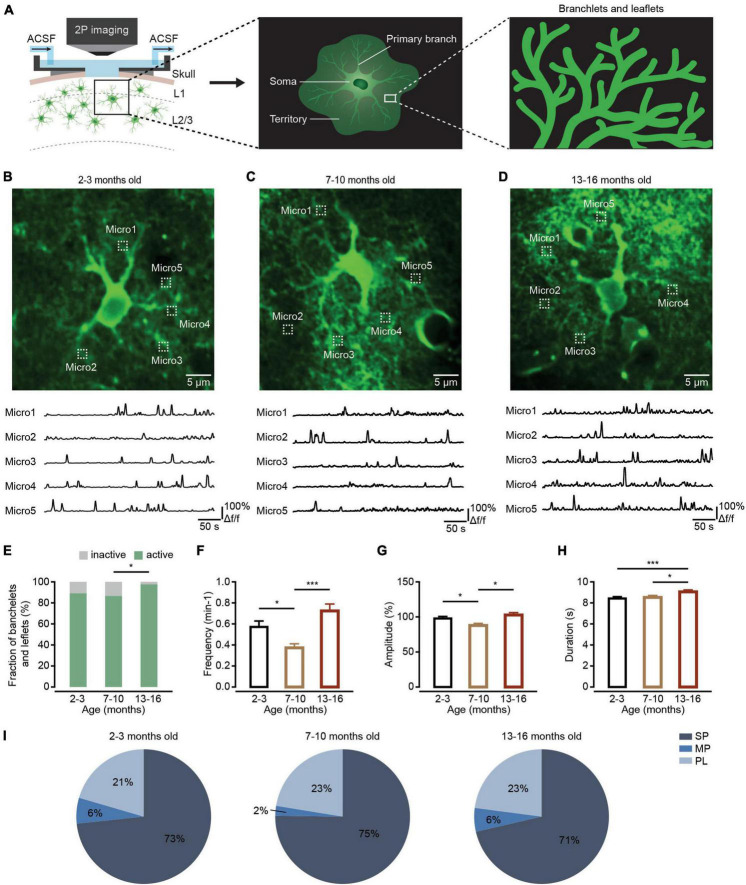
Ca^2+^ transient changes within branchlets and leaflets of cortical astrocytes during brain aging. **(A)** Schematic of two-photon Ca^2+^ imaging of astrocytic branchlets and leaflets expressing GCaMP6f. **(B–D)** Top, representative two-photon images of GCaMP6f-labeled ROIs of branchlets and leaflets in the 2–3-month-old (**B**; imaging depth, 172 μm), 7–10-month-old (**C**; imaging depth, 151 μm), and 13–16-month-old groups (**D**; imaging depth, 134 μm). Regions of branchlets and leaflets are outlined by white dashed squares. Below are example traces of spontaneous Ca^2+^ transients from each ROI. **(E)** The fraction of active branchlets and leaflets within the 2–3-month-old (*n* = 119 ROIs from six mice), 7–10-month-old (*n* = 112 ROIs from seven mice) and 13–16-month-old groups (*n* = 88 ROIs from six mice). All groups: χ^2^ = 7.6671, *P* = 0.0216; the 2-3-month-old group versus the 7–10-month-old group: *P* = 1.000; the 2–3-month-old group versus the 13–16-month-old group: *P* = 0.0529; the 7–10 month-old group versus the 13–16-month-old group: *P* = 0.0154, *P < 0.05, χ^2^-test followed by Bonferroni correction. **(F)** Bar graphs summarizing the frequencies of Ca^2+^ transients within active astrocytic branchlets and leaflets of the 2–3 month-old (*n* = 97 ROIs from six mice), 7–10-month-old (*n* = 96 ROIs from seven mice) and 13–16-month-old (*n* = 85 ROIs from six mice) groups. The 2–3 month-old group versus the 7–10-month-old group: *P* = 0.0133; the 2–3-month-old group versus the 13–16-month-old group: *P* = 0.0695; the 7–10 month-old group versus the 13–16-month-old group: *P* = 1.58 × 10^– 6^, *P < 0.05, ***P < 0.001, Kruskal–Wallis test followed by Dunn’s multiple comparisons test. **(G)** Bar graphs summarizing the amplitudes of Ca^2+^ transients within astrocytic branchlets and leaflets of the 2–3 month-old (*n* = 743 Ca^2+^ events from six mice), 7–10-month-old (*n* = 347 Ca^2+^ events from seven mice) and 13–16-month-old groups (*n* = 601 Ca^2+^ events from six mice). The 2–3-month-old group versus the 7–10-month-old group: *P* = 0.0241; the 2–3-month-old group versus 13–16-month-old group: *P* = 1.0000; the 7–10-month-old group versus the 13–16-month-old group: *P* = 0.0404, *P < 0.05, Kruskal–Wallis test followed by Dunn’s multiple comparisons test. **(H)** Bar graphs summarizing the durations of Ca^2+^ transients within astrocytic branchlets and leaflets of the 2–3-month-old (*n* = 749 Ca^2+^ events from 6 mice), 7–10-months old (*n* = 365 Ca^2+^ events from seven mice) and 13–16-month-old groups (*n* = 638 Ca^2+^ events from six mice). The 2–3-month-old group versus 7–10-month-old group: *P* = 1.0000; the 2–3-month-old group versus the 13–16-month-old group: *P* = 0.0002; the 7–10-month-old group versus the 13–16-month-old group: *P* = 0.0234, *P < 0.05, ***P < 0.001, Kruskal–Wallis test followed by Dunn’s multiple comparisons test. **(I)** Proportions of different peak types of Ca^2+^ transients within astrocytic branchlets and leaflets of the 2–3 month-old (*n* = 791 Ca^2+^ events from six mice), 7–10-month-old (*n* = 379 Ca^2+^ events from seven mice), and 13–16-month-old groups (*n* = 650 Ca^2+^ events from six mice), all groups: χ^2^ = 9.1670, *P* = 0.0571, χ^2^-test. All data are shown as the mean ± SEM. SP, singlepeaks; MP, multipeaks; PL, plateaus; Micro, micro-domain.

**FIGURE 3:**
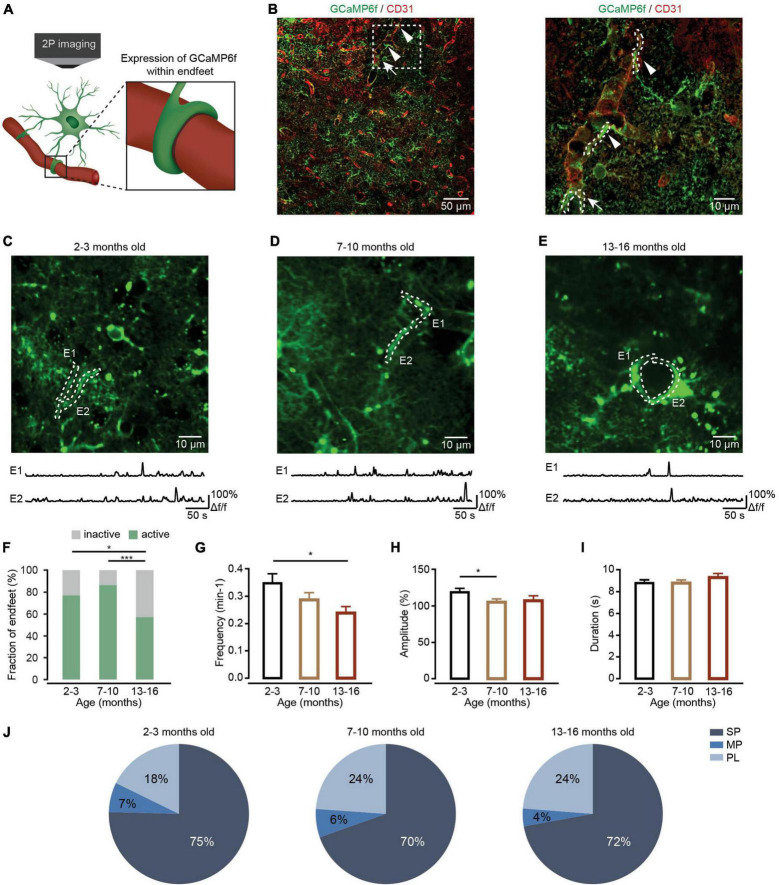
Ca^2+^ transient changes within endfeet of cortical astrocytes during brain aging. **(A)** Schematic of two-photon Ca^2+^ imaging of astrocytic endfeet expressing GCaMP6f. **(B)** Left, a representative image showing GCaMP6f (green)-labeled astrocytes and CD31 (red)-labeled blood vessels in the cortices of mice 4 weeks after AAV5-GfaABC_1_D-cytoGCaMP6f-SV40 injection. Right, a high-magnification image showing immunostaining of GCaMP6f (green) and CD31 (red) as indicated by the white-dashed box in the left panel. The arrowheads point to the sagittal sections of the endfeet. The arrow points to the coronal sections of the endfeet. **(C–E)** Top, representative two-photon images of GCaMP6f-labeled endfeet ROIs in the 2–3-month-old (**C**; imaging depth, 155 μm), 7–10-month-old (**D**; imaging depth, 107 μm), and 13–16-month-old groups (**E**; imaging depth, 113 μm). Endfeet are outlined by a white-dashed line. Below are example traces of spontaneous Ca^2+^ transients from each ROI. **(F)** The fraction of active endfeet within the 2–3-month-old (*n* = 79 ROIs from seven mice), 7–10-month-old (*n* = 89 ROIs from seven mice), and 13–16-month-old groups (*n* = 89 ROIs from seven mice). All groups: χ^2^ = 20.3088, *P* = 3.89 × 10^– 5^; the 2–3 month-old group versus the 7–10-month-old group: *P* = 0.3484; the 2–3 month-old group versus the 13–16-month-old group: *P* = 0.0189; the 7–10-month-old group versus the 13–16-month-old group: *P* = 4.35 × 10^– 5^, *P < 0.05, ***P < 0.001, χ^2^-test followed by Bonferroni correction. **(G)** Bar graphs summarizing the frequencies of Ca^2+^ transients within active astrocytic endfeet of the 2–3-month-old (*n* = 55 ROIs from seven mice), 7–10-month-old (*n* = 70 ROIs from seven mice), and 13–16-month-old (*n* = 48 ROIs from seven mice) groups. All groups: *P* = 0.0436; the 2–3 month-old-group versus 7–10-month-old group: *P* = 0.3749; the 2–3 months-old-group versus the 13–16-month-old group: *P* = 0.0389; the 7–10-month-old group versus the 13–16-month-old group: *P* = 0.7588, *P < 0.05, Kruskal–Wallis test followed by Dunn’s multiple comparisons test. **(H)** Bar graphs summarizing the amplitudes of Ca^2+^ transients within astrocytic endfeet of the 2–3-month-old (*n* = 234 Ca^2+^ events from seven mice), 7–10-month-old (*n* = 274 Ca^2+^ events from seven mice), and 13–16-month-old groups (*n* = 109 Ca^2+^ events from seven mice). The 2–3-month-old group versus the 7–10-month-old group: *P* = 0.0164; the 2–3 month-old group versus the 13–16-month-old group: *P* = 0.2502; the 7–10-month-old group versus the 13–16-month-old group: *P* = 1.0000, *P < 0.05, Kruskal–Wallis test followed by Dunn’s multiple comparisons test. **(I)** Bar graphs summarizing the durations of Ca^2+^ transients within astrocytic endfeet of the 2–3-month-old (*n* = 237 Ca^2+^ events from seven mice), 7–10-month-old (*n* = 279 Ca^2+^ events from seven mice), and 13–16-month-old groups (*n* = 120 Ca^2+^ events from seven mice). All groups: *P* = 0.1706, Kruskal–Wallis test. **(J)** Proportions of different peak types of Ca^2+^ transients within astrocytic endfeet of the 2–3-month-old (*n* = 248 Ca^2+^ events from seven mice), 7–10-month-old (*n* = 289 Ca^2+^ events from seven mice), and 13–16-month-old groups (*n* = 122 Ca^2+^ events from seven mice). All groups: χ^2^ = 4.408, *P* = 0.3540, χ^2^-test. All data are shown as the mean ± SEM. SP, singlepeaks; MP, multipeaks; PL, plateaus; E, endfoot.

Astrocytes extend highly branched processes that are coupled to the rest of the cell by narrow cytoplasmic channels (Grosche et al., 1999). These astrocytic processes are categorized as primary branches emanating from the soma (Figure 1A, right), higher-order branchlets and terminal leaflets that occupy most of the astrocytic territory (Figure 2A, right), and endfeet that contact blood vessels (Figure 3A, left; Lim et al., 2021; Semyanov and Verkhratsky, 2021). In contrast to neurons, the somatic astrocyte region is not a central signaling hub. Instead, the local Ca^2+^ transients widely distributed in astrocytic processes are thought to trigger downstream signaling cascades that modify local neuronal signaling (Semyanov et al., 2020). These local Ca^2+^ events in astrocytic processes occupy approximately 75% of the astrocyte volume (Bindocci et al., 2017) and occur independently in the soma (Lim et al., 2021). Further investigations have demonstrated that these local astrocytic Ca^2+^ transients in processes are the sites for synapse-astrocyte communications and are important for the control of synaptic transmission and plasticity (Di Castro et al., 2011). In addition, at the vascular interface, astrocytic Ca^2+^ transients are mostly restricted to individual endfeet as potential regulators of neurovascular coupling during synaptic activity (Otsu et al., 2015).

Brain aging is associated with a progressive loss of function that causes deficits in learning and memory (Verkhratsky, 2019). This degenerative progression was found to produce numerous detrimental changes in astrocytes (Matias et al., 2019), including reduced glutamate uptake (Popov et al., 2021), decreased astroglial metabolic support (Verkhratsky et al., 2021), and impaired glymphatic clearance (Kress et al., 2014). However, the effects of aging on astrocytic Ca^2+^ transients are not currently well-characterized. Some *in vitro* data indicate age-dependent remodeling of ionotropic signaling (Lalo et al., 2011) and decreased Ca^2+^ transients with aging (Gómez-Gonzalo et al., 2017; Lalo et al., 2018) occurs in astrocytes. In contrast, *in vivo* data based on Ca^2+^ analysis restricted to the soma suggest that Ca^2+^ wave activity increases in astrocytes during aging (Mathiesen et al., 2013). Until now, little was known about whether and how local Ca^2+^ signals change in astrocytic subcellular domains like branches, branchlets, leaflets, and endfeet during brain aging. Clarifying the spatial-temporal changes of these local Ca^2+^ transients during aging is a crucial step in our understanding of the astrocyte role in brain function (Zhang et al., 2021) and could reveal astrocytes as novel therapeutic targets to treat neurodegenerative diseases (Zhang et al., 2016; Yang et al., 2022).

To determine the aging-induced *in vivo* changes of Ca^2+^ transients in astrocytic processes, including the primary branches, branchlets and leaflets, and endfeet, we combined *in vivo* two-photon Ca^2+^ imaging with genetically encoded Ca^2+^ indicators (GECIs) to monitor astrocytic Ca^2+^ transients in these subcellular domains during brain aging. We found that astrocytic Ca^2+^ transients diversely change during aging within the astrocytic subcellular domains. This finding provides us with a more precise understanding of how astrocytes contribute to brain function and dysfunction during the aging process.

## Methods

### Animals

Male C57BL/6J mice were obtained from the GemPharmatech Company in Nanjing. The mice were divided into three groups according to their ages: the 2–3-month-old, 7–10-month-old, and 13–16-month-old groups. 6–9 mice were used per group, and 23 mice were used in total. Mice were maintained in a 12-h light-dark cycle at 22–25°C and 50–60% relative humidity with freely available food and water. All animal experiments were performed according to the Institutional Animal Care and Use Committee of the Third Military Medical University, China.

### Virus injection

The mice were anesthetized with 1–1.5% isoflurane in pure O_2_ and placed in stereotaxic frames. A small vertical incision was made in the skin. The part of the skull directly above the injection site was thinned using a small bone drill to allow penetration by a glass micropipette containing pAAV. To detect the characteristics of Ca^2+^ transients of astrocytes, the AAV construct (AAV5-GfaABC_1_D-cytoGCaMP6f-SV40, Cat# 52925-AAV5, Addgene) was injected with a glass micropipette at a 45° forward tilt into the somatosensory cortex [anteroposterior (AP): + 0.5 mm; mediolateral (ML): −1.56 mm, Figure 1A, left]. Two injection sites (200∼300 nl/site, without dilution: titer ≥ 7 × 10^12^ vg/mL) were applied at dorsoventral (DV): −0.7 and −0.5 mm. After the injection, the pipette was held in place for 15 min before being slowly retracted from the brain. The scalp incision was closed with tissue adhesive (Vetbond; 3M Animal Care Products); postinjection analgesics were administered to aid recovery.

### *In vivo* two-photon Ca^2+^ imaging of cortical astrocytes

Approximately 4 weeks after virus injections, *in vivo* two-photon Ca^2+^ imaging was performed as previously described (Zhang et al., 2016, 2021). Briefly, mice were anesthetized by 1–1.5% isoflurane in pure O_2_. The skin was removed after local application of xylocaine. A custom-made recording chamber was then glued to the skull with cyanoacrylate glue (UHU). A craniotomy (approximately 3 mm diameter) centered on the previous virus injection site was made by a cranial drill. During the two-photon Ca^2+^ imaging, the concentration of isoflurane decreased to 0.5–1% (breathing rates of mice were kept at 100–120 times per minute). The recording chamber was perfused with warm artificial cerebrospinal fluid (ACSF), as previously described (Zhang et al., 2021), which maintains the cell survival environment (Figure 1A, middle).

*In vivo* two-photon Ca^2+^ imaging of cortical astrocytes was performed with a custom-built two-photon microscope system (Zhang et al., 2016, 2021). Full-frame images were acquired at 20 Hz by custom-written software based on LabVIEW (National Instruments) and resampled at 5 Hz. The wavelength of the excitation laser was set at 920 nm. The average power delivered to the brain was adjusted to 15–30 mW depending on image depth to avoid phototoxicity on astrocytes.

### Immunohistochemistry

Mice were anesthetized with 1% pentobarbital (0.1 mL/g) and perfused with 4% paraformaldehyde (PFA). Free-floating coronal brain slices (40 μm thick) were obtained and stained as previously described (Qin et al., 2020; Zhang et al., 2021). In brief, brain slices were permeabilized, blocked, and incubated with primary antibodies overnight at 4°C (chicken anti-GFP, 1:500, Abcam, ab13970; rat anti-CD31,1;100, BD Biosciences, 550274; rabbit anti-S100β, 1:500, Abcam, ab41548; rabbit anti-NeuN, 1:500, Abcam, ab177487; goat anti GFAP, 1:500, Abcam, ab53554; rabbit anti AQP4, 1:500, Sigma, A5971). Sections were rinsed in PBS, followed by a 2 h incubation, in the dark at room temperature, with secondary antibodies directed against the immunoglobulins of the appropriate species coupled to AF594 and AF488 (1:500 dilution, Invitrogen). Sections were mounted with Fluorescence Mounting Medium (Fluoro-Gel II with Dapi, Electron Microscopy Sciences, Hatfield, United Kingdom). Images were acquired with a Leica TCS SP5 confocal microscope (Leica Microsystems, Wetzlar, Germany) equipped with standard filter sets and oil immersion objectives (60×/1.42 and 20×/0.85).

### Data analysis and statistics

Data analysis of two-photon Ca^2+^ imaging was completed according to our previously reported methods (Zhang et al., 2021) using LabVIEW 2014 (National Instruments), MATLAB 2014a (MathWorks), or Igor Pro 5.0 (version 6.0.3.1; WaveMetrics) in conjunction with custom-written macros. Upon displaying the maximum fluorescence projections, ROIs were manually defined for the soma, primary branches and endfeet based on high resolution (600 × 600 pixel) anatomical images (Taheri et al., 2017; Stobart et al., 2018b). Microdomains (branchlets and leaflets) were chosen at about 12–20 μm from the soma perimeter (1 × 1 μm box) according to the previous report (Taheri et al., 2017) using our custom-written macros. Astrocytic Ca^2+^ transients were expressed as relative fluorescence changes (Δf/f), corresponding to the mean fluorescence from all pixels within specified regions of interest (ROIs), as reported by our previous studies (Zhang et al., 2016, 2021). The Ca^2+^ signal for each ROI was expressed as Δf/f = (f – f_0_)/f_0_, where the fluorescence f_0_ was estimated as the 25th percentile of the entire fluorescence recording (Zhang et al., 2021). Ca^2+^ signals were defined as ‘Ca^2+^ transients’ when the maxima of the Ca^2+^ signals were above 3 × SD of the baseline that was the average of the entire 10 min Ca^2+^ signals. ROIs were classified as active if ≥ 1 Ca^2+^ transients occurred during the 10 min recording (Gómez-Gonzalo et al., 2017; Fordsmann et al., 2019). Therefore, inactive ROIs include weak Ca^2+^ signals that were smaller than 3 × SD of the baseline traces. We classified the Ca^2+^ transients as singlepeak (SP), multipeak (MP), and plateau (PL) according to a previous study (Taheri et al., 2017; Stobart et al., 2018b). SP means a signal with one clear peak, without any subsequent major oscillations or bumps; MP means a signal that has more than one peak in succession; PL means a signal with one main peak and subsequent bump or oscillation (Figure 1I). All statistics were performed in GraphPad Prism (version 8.0) and SPSS (version 25.0). The outliers of Ca^2+^ imaging data were removed according to the rule of 1.5 × interquartile range (IQR) before statistics. Normality was tested by the D’Agostino–Pearson test since the data didn’t fit the normal distribution. As such, multiple group comparisons were performed using a Kruskal–Wallis test followed by Dunn’s multiple comparisons test. Fractions of active/inactive ROIs (primary branches, branchlets and leaflets, or endfeet) and types of Ca^2+^ transients were compared with χ^2^-test or Fisher’s exact test followed by Bonferroni correction. Data were expressed as the mean ± SEM. *P* < 0.05 was considered statistically significant.

## Results

### Ca^2+^ transients within astrocytic primary branches are increased during aging

The primary branches are the astrocytic processes emanating from the soma (Figure 1A, right; Semyanov and Verkhratsky, 2021). Local Ca^2+^ elevations in the peripheral districts of astrocytic processes like branchlets and leaflets could generate propagating Ca^2+^ signals in the primary branches through the opening of endoplasmic reticulum (ER) Ca^2+^ release channels (Semyanov and Verkhratsky, 2021). Therefore, Ca^2+^ transients in the primary branches integrate the local Ca^2+^ events in the surrounding branchlets and leaflets and are important for understanding the principles of Ca^2+^ events integration within single astrocytes. First, we sought to detect aging-dependent changes to Ca^2+^ transients within the primary branches. Based on previous *in vivo* physiological studies on astrocytes (Ghosh et al., 2013; Lind et al., 2013; Kanemaru et al., 2014; Zhang et al., 2016), we injected AAV5-GfaABC_1_D-cytoGCaMP6f-SV40 into the somatosensory cortex of mice and expressed cytosolic GCaMP6f, a genetically encoded Ca^2+^ sensor, within astrocytic processes in different age groups (Figure 1A, left). Consistent with the high efficiency and specificity of GCaMP6f labeling in our previous studies (Qin et al., 2020; Zhang et al., 2021), the microinjection of this virus resulted in reliable and specific labeling of GCaMP6f within astrocytes based on immunohistochemical analysis (Supplementary Figure 1). Four weeks later, we implanted a chronic cranial window and imaged the animals by two-photon microscopy while under isoflurane anesthesia (Figure 1A, middle). Finally, we analyzed the Ca^2+^ transients within the primary branches recorded during *in vivo* two-photon imaging separately in each group (Figure 1A, right).

Figures 1B–D illustrate experiments in which we monitored the Ca^2+^ activity within primary branches of different age groups. During our analysis, we selected the primary branches from visible structures in baseline images (Figures 1B–D). The results indicated that the fractions of active primary branches did not change during the aging process (Figure 1E). However, spontaneous Ca^2+^ transients within the primary branches displayed a greater frequency (Figure 1F), a larger mean amplitude (Figure 1G), and a longer duration (Figure 1H) within active primary branches in the 13–16-month-old group than in the 2–3-month-old or 7–10 month-old groups. Based on their shape, the peaks of these Ca^2+^ transients were divided into three different classes: SP, MP, and PL (Figure 1I), which were classified according to previous studies (Taheri et al., 2017; Stobart et al., 2018b). Statistical analysis indicated that the percentage of SP decreased, while the percentages of MP increased during aging within active primary branches (Figure 1J).

### Ca^2+^ transients within astrocytic branchlets and leaflets first decreased and then increased during aging

Branchlets and leaflets are higher-order and terminal processes surrounding the primary branches. Most cannot be resolved with diffraction-limited optical imaging, appear as a spongiform cloud, and occupy most of the astrocyte territory (Figure 2A, middle and right) (Semyanov and Verkhratsky, 2021). Most of spontaneous Ca^2+^ activity occurs as localized transient elevations of [Ca^2+^]i are detected in branchlets and leaflets (Lim et al., 2021). These local Ca^2+^ transients are often triggered by neurotransmitters and modify local neuronal signaling (Semyanov et al., 2020).

We then detected aging-dependent changes to Ca^2+^ transients within astrocytic branchlets and leaflets. Using the above-described methods, astrocytic branchlets and leaflets were labeled by cytosolic GCaMP6f 4 weeks after microinjection of AAV5-GfaABC_1_D-cytoGCaMP6f-SV40 into the somatosensory cortex. The Ca^2+^ transients within astrocytic branchlets and leaflets were recorded using *in vivo* two-photon microscopy in each group (Figure 2A). According to the previous report (Taheri et al., 2017), Ca^2+^ activities in microdomains (1 × 1 μm box) at approximately 12–20 μm from the soma perimeter were applied to represent Ca^2+^ transients within the branchlets and leaflets (Figures 2B–D). We found that the fraction of active branchlets and leaflets increased during aging (Figure 2E). The frequency (Figure 2F) and amplitude (Figure 2G) of spontaneous Ca^2+^ transients within the active branchlets and leaflets decreased in the 7–10-month-old group and increased in 13–16-month-old group, while the duration of Ca^2+^ transients within the active branchlets and leaflets was unchanged in the 7–10-month-old group and increased in 13–16-month-old group (Figure 2H). In addition, consistent with the changes of the frequency and amplitude during aging, the percentage of MP type of Ca^2+^ transients decreased in the 7–10-month-old group and increased in 13-16-month-old group (all groups: *P* = 0.0571, χ^2^-test; Figure 2I).

### Ca^2+^ transients within astrocytic endfeet decreased during aging

Endfeet are the astrocytic domains closest to blood vessels and envelop these vessels. Ca^2+^ transients within the astrocytic endfeet are important for regulating neurovascular coupling (Otsu et al., 2015). To determine whether aged astrocytes show differences in the characteristics of Ca^2+^ transients within endfeet, we expressed cytosolic GCaMP6f in astrocytic endfeet with injections of AAV5-GfaABC_1_D-cytoGCaMP6f-SV40. Four weeks after virus injection, Ca^2+^ transients within astrocytic endfeet were recorded by *in vivo* two-photon microscopy in anesthetized mice of different age groups (Figure 3A). Immunohistochemistry data shows that astrocytic endfeet expressing GCaMP6f envelop CD31-positive blood vessels (Figure 3B). During data analysis, endfeet regions were selected from visible structures in baseline images according to the previous study (Stobart et al., 2018a,b; Figures 3C–E). Statistical results demonstrated that aging decreased the fractions of active endfeet (Figure 3F). In addition, the frequency and amplitude of Ca^2+^ transients decreased during aging within active endfeet (Figures 3G,H). However, the duration of astrocytic Ca^2+^ transients (Figure 3I) and the percentages of Ca^2+^ transient types (all groups: *P* = 0.3540, χ^2^-test; Figure 3J) were not affected by aging within active endfeet.

**FIGURE 4:**
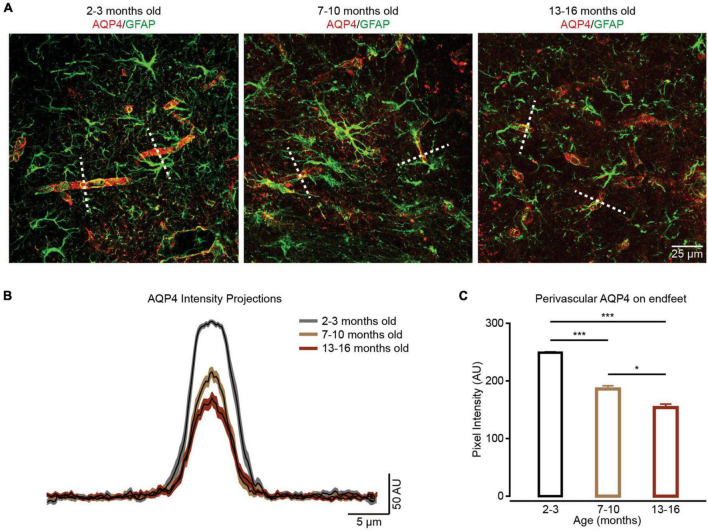
The localization of perivascular AQP4 on endfeet is lost during brain aging. **(A)** In contrast to the well-maintained localization of perivascular AQP4 (red) on endfeet (perivascular GFAP labeled green structure) in the 2–3-month-old group (left panel), perivascular AQP4 localization becomes more dispersed in the 7–10-month-old and 13–16-month-old groups (middle and right panel). **(B)** AQP4 immunofluorescence was evaluated in linear regions of interest (dashed lines, **A**) extending outward from vessels. **(C)** Bar graph summarizing measurement of perivascular AQP4 expression on endfeet. Compared with the 2–3-month-old group (*n* = 47 vessels from four mice), perivascular AQP4 expression on endfeet decreased in the 7–10-month-old group (*n* = 40 vessels from four mice) and 13–16-month-old group (*n* = 45 vessels from four mice); the 2–3-month-old group versus 7–10-month-old group: *P* = 6.67 × 10^– 10^; the 2–3-month-old group versus the 13–16-month-old group: *P* = 4.1959 × 10^– 20^; the 7–10-month-old group versus the 13–16-month-old group: *P* = 0.0359, *P < 0.05, ***P < 0.001, Kruskal–Wallis test followed by Dunn’s multiple comparisons test. All data are shown as the mean ± SEM.

Aquaporin-4 (AQP4) is the predominant water channel localized in the astrocytic endfeet and mediates water transport into the brain parenchyma (Vandebroek and Yasui, 2020). A previous study suggested that AQP4 is involved in regulating the Ca^2+^ transients within the endfeet (Thrane et al., 2011). We also detected the expressions of AQP4 in endfeet in different groups by immunohistochemistry (Figure 4A). Similar to our previous report (Yang et al., 2022), the current results indicated that AQP4 expression in endfeet decreased during brain aging (Figures 4B,C), which could decrease Ca^2+^ transients within endfeet (Thrane et al., 2011).

In addition, changes in Ca^2+^ transients within the somata of astrocytes were assessed during brain aging. Data indicated that aging did not affect the characteristics (frequency, amplitude, and duration) and proportions of Ca^2+^ transient types within astrocytic somata (all groups: *P* = 0.7468, Fisher’s exact test; Supplementary Figure 2). This result suggests that astrocytic processes and endfeet are more sensitive to aging-induced chronic stress than somata.

## Discussion

Arborization of astrocytes is classified into (i) primary branches emanating from soma; (ii) branchlets and leaflets that occupy most of the astrocyte territory also known as an anatomic domain; and (iii) endfeet that are specialized extensions of astrocytic branches contacting and plastering blood vessel (Semyanov and Verkhratsky, 2021). Most of spontaneous Ca^2+^ transients reside in the above astrocyte subcellular domains (Lim et al., 2021), heterogeneously occur in an individual astrocyte (Bindocci et al., 2017), and are independent of somatic Ca^2+^ signals (Lim et al., 2021). These local Ca^2+^ transients in peripheral processes and endfeet of astrocytes are important for their normal functions: in primary branches, Ca^2+^ transients, representing the signal communications between soma and processes, indicate the intrinsic principles of Ca^2+^ signal integration in an individual astrocyte (Semyanov et al., 2020); in branchlets and leaflets, Ca^2+^ transients modify local neuronal signaling and control synaptic transmission (Lim et al., 2021); in endfeet, Ca^2+^ transients contribute to the regulation of neurovascular coupling (Otsu et al., 2015). Therefore, deciphering the complex and heterogeneous “Ca^2+^ language” in these subcellular domains is essential for defining how astrocytes contribute to brain function and dysfunction.

Aging reduces the functional capacity of astrocytes, including reduced K^+^ buffering and glutamate clearance (Popov et al., 2021), decreased astroglial gap junctional coupling, and impaired astroglial metabolism (Jiang and Cadenas, 2014). In aging brains, astrocytes exhibit ionic excitability mediated by changes in the intracellular concentration of ions (Verkhratsky, 2019) and show peculiar reorganization of astrocytic Ca^2+^ signals (Popov et al., 2021). This aging-induced aberrant Ca^2+^ signaling impacts synaptic plasticity and affects long-term potentiation in hippocampal synapses (Popov et al., 2021). Importantly, it has been suggested that changes in Ca^2+^ homeostasis and Ca^2+^ signaling are a general mechanism of neural cell aging, which was formalized in a “calcium hypothesis of brain aging” (Khachaturian, 1987; Verkhratsky and Toescu, 1998). More detailed analyses revealed that aging seems to lead to enhanced sensitivity of the brain to changes in [Ca^2+^]i. Aged brain cells could be more vulnerable because of a decreased ability to down-regulate [Ca^2+^]i (Müller et al., 1996). Considering the functional importance of subcellular Ca^2+^ signaling in astrocytes, we studied whether and how these local Ca^2+^ transients change during aging to further illustrate the role of astrocytes in brain aging.

The present study found that aging alters characteristics of Ca^2+^ transients in all kinds of subcellular domains, including primary branches, branchlets and leaflets, and endfeet. However, these age-dependent changes differ between these subcellular domains, suggesting there are distinct aging-induced degenerative mechanisms occurring in separate local regions within an individual astrocyte.

In primary branches, Ca^2+^ transients are initiated by the release of Ca^2+^ from the ER through the inositol 1,4,5-trisphosphate receptor type 2 (IP3R2) in response to stimulation of metabotropic receptors (Shigetomi et al., 2013; Kanemaru et al., 2014; Lim et al., 2021). Previous studies have indicated that astrocytes show an age-dependent increase in oxidative metabolism and reactive oxygen species (ROS) (Jiang and Cadenas, 2014). In addition, ROS causes oxidation of the IP3R2 and sensitization of Ca^2+^ release to promote cytoplasmic Ca^2+^ oscillations (Bánsághi et al., 2014). ROS also activates ryanodine receptor Ca^2+^ release channels in ER and increases the number of Ca^2+^ transients (Prosser et al., 2013). Therefore, the enhanced oxidative stress during physiological aging could increase Ca^2+^ transients within primary branches of astrocytes. Furthermore, the surface-to-volume ratio (SVR) sets the threshold for the generation of spreading Ca^2+^ events and determines the probability of Ca^2+^ transient initiation (Rungta et al., 2016; Wu et al., 2019). The primary branches have higher SVR than the somata. As such, Ca^2+^ entering the primary branch will produce larger increases in Ca^2+^ concentration, which are more likely than increases in somatic Ca^2+^ concentration to reach the threshold for amplification by Ca^2+^-dependent Ca^2+^ release (Semyanov et al., 2020). This could be why aging caused Ca^2+^ transient changes in primary branches, but not in somata (Figure 1 and Supplementary Figure 2).

In branchlets and leaflets, Ca^2+^ transients are generated by Ca^2+^ release from ER and mitochondria in branchlets and Ca^2+^ entry through the plasma membrane *via* sodium/calcium exchanger (NCX), ionotropic receptors or channels in leaflets (Semyanov et al., 2020). In this study, we found that the Ca^2+^ transients within branchlets and leaflets first decreased in 7–10-months-old mice, and then increased in 13–16-months-old mice (Figure 2). The decreased Ca^2+^ transients in 7–10-months-old mice could be caused by the age-dependent remodeling of ionotropic signaling in astrocytes (Lalo et al., 2011). Certain kinds of ionotropic receptors peak in young adults (3-months-old) and decrease during aging (Lalo et al., 2011). The age-dependent increase in oxidative metabolism in astrocytes (Jiang and Cadenas, 2014) could induce the following increased Ca^2+^ transients in 13–16-month-old mice. It has been shown that increased ROS levels not only enhance Ca^2+^ release from ER (Prosser et al., 2013), but also promote Ca^2+^ efflux from mitochondria *via* the mitochondrial permeability pore (mPTP) (Agarwal et al., 2017). Additionally, the increased Ca^2+^ transients in branchlets and leaflets during aging could be caused by decreases in the partial pressure of oxygen (P_*O*2_) in the aged brain (Mathiesen et al., 2013). It has been reported that lowering P_*O*2_ in the aged brain resulted in increased cytosolic NADH that induced pronounced increase in Ca^2+^ signaling in astrocytes (Requardt et al., 2012).

Previous studies have found that Ca^2+^ transients within the endfeet are involved in the modulation of neurovascular coupling (Otsu et al., 2015). These Ca^2+^ transients are partly triggered by AQP4-mediated water influx, which promotes ATP release and activation of P2 purinergic receptors (Thrane et al., 2011). In a normal physiological state, the expression of AQP4 is localized to the astrocytic endfeet. However, a previous study (Valenza et al., 2019) and our data (Figure 4) both indicated that the localization of perivascular AQP4 on endfeet is lost during brain aging. Therefore, the decreased Ca^2+^ transients within the endfeet (Figure 3) could be caused by decreased expression of AQP4 on endfeet, which results in decreased water influx, the release of ATP, and activation of P2 purinergic receptors. At the same time, Ca^2+^ transients within the endfeet are also mediated by mitochondria (Göbel et al., 2020) and GABAA receptors (Lind et al., 2018). Therefore, the aging-induced impairment of mitochondrial Ca^2+^ uptake (Göbel et al., 2020) and decreases in ionotropic receptor expression (Lalo et al., 2011) can also contribute to decreases in Ca^2+^ transients observed within endfeet. In addition, according to previous studies, IP3R2-mediated Ca^2+^ transients are present within branches and branchlets (Shigetomi et al., 2013; Kanemaru et al., 2014), but absent within endfeet (Bonder and McCarthy, 2014), which could lead to the diverse impacts of aging on Ca^2+^ transients within these subcellular domains.

Apart from that, both the age-dependent morphological changes and astrocytic network alterations may induce the aberrant astrocytic Ca^2+^ transients at subcellular domains during brain aging. Previous studies indicated that aging reduces cellular surface area and morphological complexity of astrocytes (Popov et al., 2021; Yang et al., 2022). These age-dependent morphological changes are correlated to spatiotemporal reorganization and increased duration of Ca^2+^ transients in old astrocytes (Popov et al., 2021). In addition, astrocytic network alterations have been reported in neurodegenerative disorders, which induced the elevated resting Ca^2+^ and more frequent Ca^2+^ transients in astrocytic network (Kuchibhotla et al., 2009; Delekate et al., 2014).

There are three different Ca^2+^ transient types based on their shapes: singlepeaks, multipeaks, and plateaus (Figure 1I). Different Ca^2+^ transient types indicate diverse mechanisms of signaling (Stobart et al., 2018b). In this study, we found that the percentages of Ca^2+^ transient types changed with aging in primary branches (Figure 1J) and branchlets and leaflets (Figure 2I), but remained unchanged in endfeet (Figure 3J). This suggests that, aging differentially affects the mechanisms of Ca^2+^ signaling in astrocyte subcellular domains. Specifically, it suggested that the IP3-mediated release of ER calcium stores contributed to singlepeaks and multipeaks (Stobart et al., 2018b). As such, aging-changed singlepeak and multipeak proportions further indicated that ROS-mediated IP3R2 oxidation (Bánsághi et al., 2014) could be the main mechanism of Ca^2+^ transient changes during aging within primary branches (Figure 1J), branchlets and leaflets (Figure 2I).

Our study indicated that there was no change of Ca^2+^ transients within astrocytic somata during brain aging (Supplementary Figure 2), suggesting branches, branchlets and leaflets, or endfeet were more sensitive to aging-induced oxidative stress than somata. Furthermore, previous studies indicated that astrocytic Ca^2+^ transients remained stable (Gómez-Gonzalo et al., 2017) or declined (Lalo et al., 2018) during aging, which could be caused by the *in vitro* methods and chemical calcium indicators used in these experiments.

Altogether, our *in vivo* results demonstrate that aging alters the characteristics of Ca^2+^ transients within astrocyte subcellular domains. However, these changes in Ca^2+^ transients are heterogeneous in the subcellular domains, which indicates that the aging-induced degenerative mechanisms differ at the subcellular level in astrocytes. This finding provides us a better understanding of how astrocytes contribute to brain dysfunction during aging and other neural degenerative diseases, like Alzheimer’s disease and Parkinson’s disease.

## Data availability statement

The raw data supporting the conclusions of this article will be made available by the authors, without undue reservation.

## Ethics statement

The animal study was reviewed and approved by the Institutional Animal Care and Use Committee of the Third Military Medical University.

## Author contributions

LF and KZ designed the work. FD, SL, RL, ZY, YH, SY, QD, JZ, JLy, ZZ, MH, HW, JLi, CY, YW, MG, SC, and HJ performed the main experiments. FD, ZY, MH, and HW completed the virus injections. FD, RL, YH, and JZ performed the two-photon Ca^2+^ image. FD, ZY, JLi, CY, YW, and MG performed the immunohistochemistry. FD, SL, QD, JLy, ZZ, HJ, and XL performed the data analysis. XC, XL, LF, and KZ wrote the manuscript with input from all co-authors. All authors contributed to the article and approved the submitted version.

## Supplementary material

Supplementary Figure 1 Specific and efficient expression of GCaMP6f in cortical astrocytes 4 weeks after AAV5-GfaABC_1_D-cytoGCaMP6f-SV40 injection. **(A)** Representative image showing most of the GCaMP6f (green)-labeled astrocytes were S100β (red) positive cells. **(B)** High-magnification image showing immunostaining of GCaMP6f (green) and S100β (red) as indicated by the white-dashed box in the left panel. **(C)** Representative image showing none of the GCaMP6f-labeled astrocytes were stained with NeuN.

Supplementary Figure 2 No changes to Ca^2+^ transients within the somata of cortical astrocytes during brain aging. **(A)** The fraction of active somata within the 2–3-month-old (*n* = 126 ROIs from seven mice), 7–10-month-old (*n* = 126 ROIs from seven mice), and 13–16-month-old groups (*n* = 116 ROIs from seven mice), all groups: χ^2^ = 1.2468, *P* = 0.5261, χ^2^-test. **(B)** Bar graphs summarizing the frequencies of Ca^2+^ transients within active astrocytic somata of the 2–3-month-old (*n* = 48 ROIs from seven mice), 7–10-month-old (*n* = 53 ROIs from seven mice), and 13–16-month-old (*n* = 42 ROIs from seven mice) groups. All groups: *P* = 0.3233, Kruskal–Wallis test. **(C)** Bar graphs summarizing the amplitudes of Ca^2+^ transients within astrocytic somata of the 2–3-month-old (*n* = 134 Ca^2+^ events from seven mice), 7–10-month-old (*n* = 124 Ca^2+^ events from seven mice), and 13–16-month-old (*n* = 83 Ca^2+^ events from seven mice) groups. All groups: *P* = 0.5258, Kruskal–Wallis test. **(D)** Bar graphs summarizing the durations of Ca^2+^ transients within astrocytic somata of the 2–3-month-old (*n* = 133 Ca^2+^ events from seven mice), 7–10-month-old (*n* = 117 Ca^2+^ events from seven mice), and 13–16-month-old (*n* = 80 Ca^2+^ events, seven mice) groups. All groups: *P* = 0.0920, Kruskal–Wallis test. **(E)** Proportions of different peak types of Ca^2+^ transients within somata of the 2–3-month-old (*n* = 138 Ca^2+^ events from seven mice), 7–10-month-old (*n* = 128 Ca^2+^ events from seven mice), and 13–16-month-old groups (*n* = 87 Ca^2+^ events from seven mice). All groups: *P* = 0.7468, Fisher’s exact test. All data are shown as the mean ± SEM. SP, singlepeaks; MP, multipeaks; PL, plateaus; S, soma.
