# Supplementary figures and images for: Astrocytes exhibit diverse Ca2+ changes at subcellular domains during brain aging
Source: Front Aging Neurosci. 2022 Oct 28;14:1029533. doi: 10.3389/fnagi.2022.1029533 (PMC9650392; doi:10.3389/fnagi.2022.1029533)

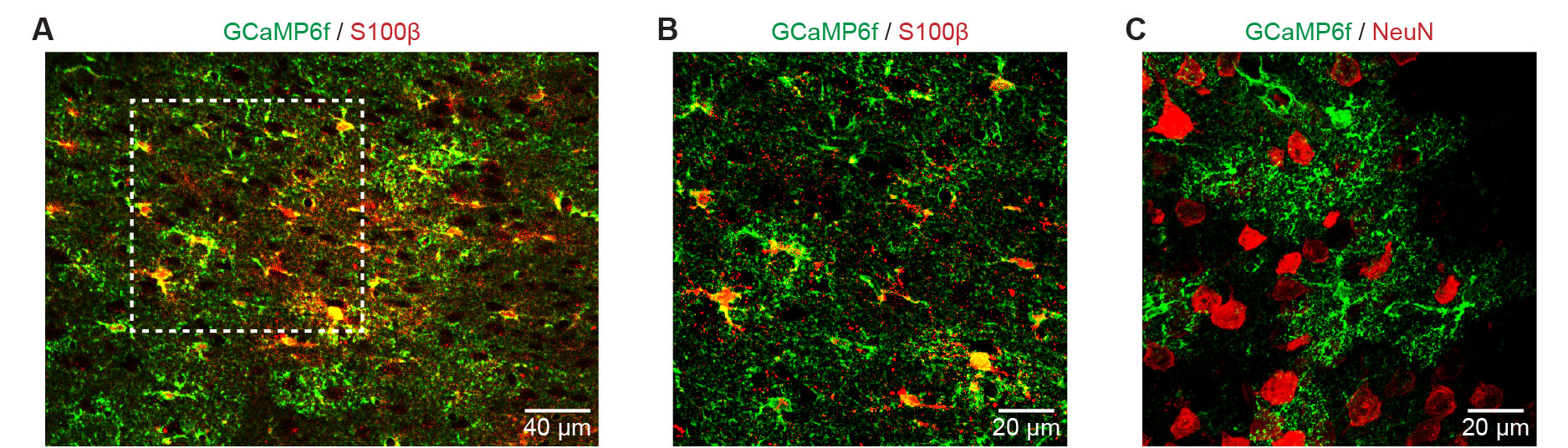

Supplement: Supplementary Figure 1 — Specific and efficient expression of GCaMP6f in cortical astrocytes 4 weeks after AAV5-GfaABC1D-cytoGCaMP6f-SV40 injection. (A) Representative image showing most of the GCaMP6f (green)-labeled astrocytes were S100β (red) positive cells. (B) High-magnification image showing immunostaining of GCaMP6f (green) and S100β (red) as indicated by the white-dashed box in the left panel. (C) Representative image showing none of the GCaMP6f-labeled astrocytes were stained with NeuN. [file Image_1.JPEG]

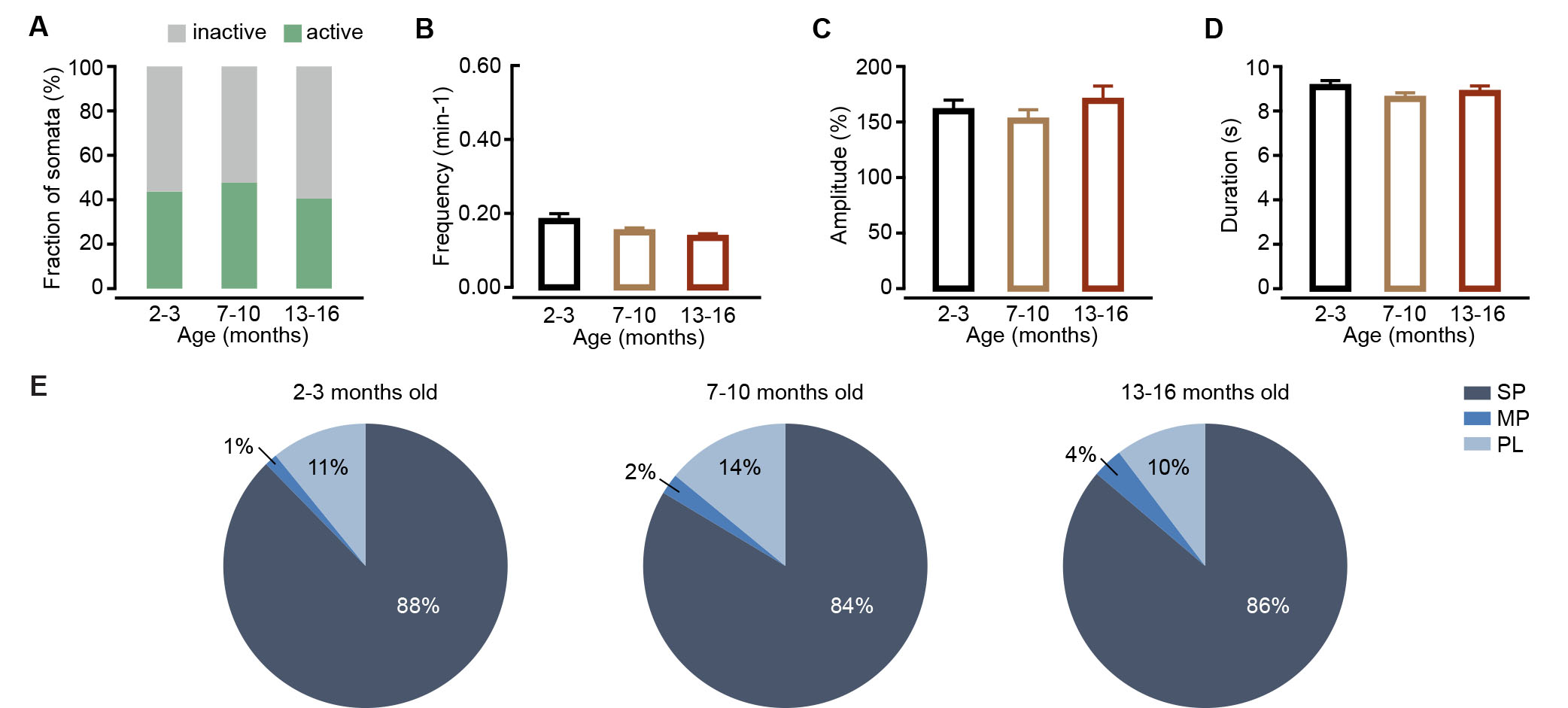

Supplement: Supplementary Figure 2 — No changes to Ca2+ transients within the somata of cortical astrocytes during brain aging. (A) The fraction of active somata within the 2–3-month-old (n = 126 ROIs from seven mice), 7–10-month-old (n = 126 ROIs from seven mice), and 13–16-month-old groups (n = 116 ROIs from seven mice), all groups: χ2 = 1.2468, P = 0.5261, χ2-test. (B) Bar graphs summarizing the frequencies of Ca2+ transients within active astrocytic somata of the 2–3-month-old (n = 48 ROIs from seven mice), 7–10-month-old (n = 53 ROIs from seven mice), and 13–16-month-old (n = 42 ROIs from seven mice) groups. All groups: P = 0.3233, Kruskal–Wallis test. (C) Bar graphs summarizing the amplitudes of Ca2+ transients within astrocytic somata of the 2–3-month-old (n = 134 Ca2+ events from seven mice), 7–10-month-old (n = 124 Ca2+ events from seven mice), and 13–16-month-old (n = 83 Ca2+ events from seven mice) groups. All groups: P = 0.5258, Kruskal–Wallis test. (D) Bar graphs summarizing the durations of Ca2+ transients within astrocytic somata of the 2–3-month-old (n = 133 Ca2+ events from seven mice), 7–10-month-old (n = 117 Ca2+ events from seven mice), and 13–16-month-old (n = 80 Ca2+ events, seven mice) groups. All groups: P = 0.0920, Kruskal–Wallis test. (E) Proportions of different peak types of Ca2+ transients within somata of the 2–3-month-old (n = 138 Ca2+ events from seven mice), 7–10-month-old (n = 128 Ca2+ events from seven mice), and 13–16-month-old groups (n = 87 Ca2+ events from seven mice). All groups: P = 0.7468, Fisher’s exact test. All data are shown as the mean ± SEM. SP, singlepeaks; MP, multipeaks; PL, plateaus; S, soma. [file Image_2.JPEG]
